# Approaches to Managing Autoimmune Cytopenias in Novel Immunological Disorders with Genetic Underpinnings Like Autoimmune Lymphoproliferative Syndrome

**DOI:** 10.3389/fped.2015.00065

**Published:** 2015-07-21

**Authors:** V. Koneti Rao

**Affiliations:** ^1^ALPS Clinic, Laboratory of Clinical Infectious Diseases, Division of Intramural Research, National Institute of Allergy and Infectious Diseases, National Institutes of Health, Department of Health and Human Services, Bethesda, MD, USA

**Keywords:** cytopenias, lymphoproliferative disorders, genetic disease, autoimmune lymphoproliferative syndrome, autoimmune diseases

## Abstract

Autoimmune lymphoproliferative syndrome (ALPS) is a rare disorder of apoptosis. It is frequently caused by mutations in FAS (*TNFRSF6*) gene. Unlike most of the self-limiting autoimmune cytopenias sporadically seen in childhood, multi lineage cytopenias due to ALPS are often refractory, as their inherited genetic defect is not going to go away. Historically, more ALPS patients have died due to overwhelming sepsis following splenectomy to manage their chronic cytopenias than due to any other cause, including malignancies. Hence, current recommendations underscore the importance of avoiding splenectomy in ALPS, by long-term use of corticosteroid-sparing immunosuppressive agents like mycophenolate mofetil and sirolimus. Paradigms learnt from managing ALPS patients in recent years is highlighted here and can be extrapolated to manage refractory cytopenias in patients with as yet undetermined genetic bases for their ailments. It is also desirable to develop international registries for children with rare and complex immune problems associated with chronic multilineage cytopenias in order to elucidate their natural history and long-term comorbidities due to the disease and its treatments.

## Introduction

Autoimmune lymphoproliferative syndrome (ALPS)^1^ is a disorder of the immune system due to defective Fas-mediated apoptosis (1). Mutations in FAS (TNFRSF6) gene commonly cause what is known as ALPS-FAS. Patients with undetermined genetic defect are classified as ALPS-U. ALPS presents in childhood with lymphadenopathy, hypersplenism, and multilineage cytopenias. Many patients experience a lessening of symptoms in adulthood, most likely due to age-associated immune modulation. ALPS due to germline FAS mutations is inherited in an autosomal dominant manner (2, 3), however somatic FAS mutations limited to circulating lymphocyte subsets leading to clinical manifestations of ALPS have also been reported over the years (4–6). Of the estimated 1000 ALPS patients reported thus far worldwide, over 300 participate in studies at the NIH Clinical Center. Over many years of follow-up, the general morbidity attributable to ALPS includes the frequent need for splenectomy and the risks of overwhelming post-splenectomy infection (OPSI) leading to sepsis, recurrent and chronic multilineage cytopenias, and development of lymphomas (7, 8). Among ALPS-FAS patients, standardized incidence ratio values are 149 and 61 times higher for Hodgkin and non-Hodgkin lymphoma, respectively compared to US general population. However, historically more ALPS-FAS patients have died due to overwhelming sepsis following splenectomy to manage their cytopenias than due to any other cause, including malignancies and recently ALPS patients have been noted to have a unique IgM-mediated immune surveillance defect following exposure to pneumococci (7–9). Among the 66 splenectomized patients in our recently published cohort, 41% (*n* = 27) had multiple episodes of pneumococcal sepsis, and 6 of them died. The likelihood of cytopenia relapse after splenectomy was 30% by 4 years and exceeded 70% by 20 years thus underscoring the futility of splenectomy itself as an intervention. Hence, current recommendations based on published literature are highlighted here (10, 11). Splenectomy should be avoided in ALPS patients by using corticosteroid-sparing immunosuppressive agents like mycophenolate mofetil (MMF) and sirolimus for the long-term management of their concurrent or sequential cytopenias.

Treatment interventions that include ruling out malignancies are indicated in ALPS patients at any age when they present with clinically significant and refractory cytopenias. Clinicians caring for these patients need to be familiar with their disease course, vigilant for the complications including lymphoma development, and wary of the long-term toxicity and accompanying morbidity of any pharmacological intervention they might contemplate to undertake. It is hoped that paradigms learnt during the last two decades from diagnosing and managing ALPS as highlighted here can be applicable to other novel inherited immune disorders presenting with nodal and extranodal lymphoproliferation, infection, end organ damage as well as T- and B-cell dysfunction leading to many autoimmune complications including cytopenias (10, 12–16).

## Diagnosis and Management of Cytopenias in ALPS

Cytopenias due to underlying immunological disorders like secondary chronic immune thrombocytopenia (ITP) seen in SLE patients have been known to present their own unique clinical challenges which includes establishing an early diagnosis, ruling out malignancies, and minimizing end organ damage while rendering long-term care (17, 18). Diagnosis of ALPS should be based on published criteria, which requires presence of lymphadenopathy, splenomegaly, and increased (>2.5%) CD4-/CD8-double negative TCR alpha/beta+ T cells in peripheral blood circulation, the signature cells of ALPS. Other biomarkers including increased serum vitamin B12, IgG, IL10, and very low HDL provide helpful clues to search for a FAS mutation leading to a diagnosis of ALPS-FAS, by far the most well described and common subtype of ALPS seen in nearly two-thirds of the cohorts worldwide (19–22). We recently summarized our experience related to cytopenias in 150 patients with ALPS-FAS with a median follow-up of 13.5 years (8). Recurrent multilineage cytopenias were common, seen in two-thirds (104/150) of them. Their median age of initial presentation was 5.6 years with a range of age of disease onset from 1 to 53 years. There were no gender differences. Single lineage, bilineage, and trilineage cytopenias were seen in 21, 23, and 25% of them, respectively. Eighty-eight patients were treated long-term for cytopenias, but their need for treatment frequency appeared to decline with age. Pulse doses of corticosteroids, often the most effective immediate intervention was given to 90% of them. However, most patients required multiple agents that included long-term (>1 year) immunosuppression using MMF, rapamycin (sirolimus), intravenous immunoglobulin, rituximab, vincristine, cytoxan, hydroxychloroquine, 6MP, and WinRho, although one individual developed severe hemolysis after WinRho. This prompted us not to use WinRho for ALPS patients thereafter, as many of them are already Coomb’s DAT positive without overt hemolytic anemia. In the following section, an updated narrative of the long-term experience so far using immunosuppressive agents including MMF and sirolimus for managing cytopenias and other autoimmune complications in ALPS patients is presented (10, 16, 23).

Corticosteroids and many of the immunosuppressive drugs including azathioprine, cyclosporine, or MMF, fail to reliably shrink the spleen or lymph nodes in ALPS patients. Actually, no intervention is usually indicated in many of these patients unless and until they present with clinically significant cytopenias. These can be due to splenic sequestration, bone marrow infiltration with non-malignant lymphoproliferation consisting of clustering islands of T and B lymphocytes, and/or autoimmune peripheral destruction of blood cells in the reticuloendothelial system. Hence, systemic symptoms such as fever, fatigue, weight loss, loss of appetite, and/or sudden focal lymph node enlargement should be carefully evaluated and lymphoma should be ruled out in the clinical context prior to embarking on definitive treatments for cytopenias using immunosuppressive agents. The initial management autoimmune cytopenias including autoimmune hemolytic anemia (AIHA), immune-mediated thrombocytopenia, autoimmune neutropenia in ALPS patients is not much different from management of sporadic immune cytopenias in other patient populations (24). Following principles are outlined specifically as a guideline for ALPS patients with refractory cytopenias based on our experience derived from managing them over the last 15 years. Many patients included here and summarized in Table 1 have been exposed to multiple agents sequentially as described below.

**Table 1:** **Cytopenia treatment outcomes in ALPS patients**.

Patients	MMF (*n* = 64)	Sirolimus (*n* = 14)	Rituximab (*n* = 26)	Hydroxychloroquine (*n* = 4)	TPO mimetics (Eltrombopag and Nplate) (*n* = 4)
ALPS-FAS	45	12	20	4	3
ALPS-U	19	2	6		1
Gender ratio (male/female)	49:15	10:4	20:6	2:2	3:1
Median age at start of medication (range)	10 years (0.5–34 years)	2 years (1–37 years)	13 years (1–47 years)	17 years (12–30 years)	19 years (9–20 years)
Median duration of follow-up on medication (range, years)	4.5 years (0.5–l5 years)	(0.5–6 years)	NA	3 years (0.5–7 years)	2.25 (0.5–4 years)
Long-term responders	44	10	16^a^	2	2
Non-responders (requiring other interventions)	20	4	10	2	2

*^a^Remission lasted at least 6 months after one course of rituximab 375 mg/M2 weekly × 4*.

### Immune suppression with corticosteroids

High-doses of pulse therapy should be initiated with intravenous methylprednisolone (5–10 mg/kg × 7–10 days). This should be followed by oral prednisone (1–2 mg/kg) as a maintenance therapy that can be tapered over a period of several weeks (8–12 weeks). Higher doses of intravenous methylprednisolone (up to 30 mg/kg per day for 1–3 days) may have to be used in for severe and refractory cytopenias (e.g., very low hemoglobin of <5 g/dL). Usual steroid-related comorbidities including hypertension, hyperglycemia, cataracts, osteopenia, and avascular necrosis of the hip as well as Cushingoid body habitus has prompted us to avoid their long-term use.

### Intravenous immunoglobulin G

Intravenous immunoglobulin G (1-2g/kg/day × 2 days) is usually given concomitantly with pulse dose IV methylprednisolone. This helps some patients with severe AIHA or ITP as IVIG uptake by the reticuloendothelial system in the spleen, liver, and bone marrow reduces antibody-mediated red cell and/or platelet destruction. This also sustains packed red blood cell transfusion support toward relief of anemia in the setting of active ongoing hemolysis.

### Role of granulocyte colony stimulating factor in isolated neutropenia

Twice or thrice weekly, very low dose G-CSF (1–2 mcg/kg) given subcutaneously may benefit some ALPS patients. However, G-CSF should only be reserved for treating isolated neutropenia with associated infections in patients without significant splenic enlargement in order to avoid possibility of further G-CSF induced splenic enlargement and rupture. It is always prudent to check CBCs to rule out neutropenia and provide antibiotic prophylaxis as needed before dental work and other elective surgeries in some of these patients with significant neutropenia.

### Mycophenolate mofetil

We described our experience using MMF for treating chronic cytopenias in 13 children with ALPS in 2005 (13). MMF, given twice daily orally (600 mg/m2 per dose), has enabled us to avoid chronic steroid usage in many patients with ALPS-associated refractory cytopenias over the last 15 years. We have used MMF in 64 ALPS patients (median age 10 years with a range of 6 months to 15 years) as ongoing long-term steroid sparing immune suppression. These patients have had a median follow-up of 4.5 years (range, 6 months to 15 years) in our clinic. Most of them have chronic and refractory cytopenias. Some of them also have other autoimmune manifestations, such as uveitis, hepatitis, glomerulonephritis, and infiltrative pulmonary lesions. Notably 18 of them have had their cytopenias relapse following splenectomy prior to commencing treatment with MMF. Most patients (60/64) responded to MMF. Their response was predicated by adequate hemoglobin, neutrophil, and platelet counts. In responders, we could reduce dose or stop other immunosuppressive agents for at least 1 year. However, 20 of them required other therapies (including switch to sirolimus in 7 of them with hypersplenism) later on as their cytopenias became more refractory. One ALPS-FAS patient developed Hodgkin’s lymphoma, but he continues to require MMF for cytopenia for the last 9 years; another patient with ALPS-U while on MMF for 8 years underwent bone marrow transplantation for acute leukemia and he is no longer requiring MMF as his cytopenias have resolved. One ALPS-FAS patient after a period of response to MMF for 5 years had overwhelming AIHA and died. MMF has also allowed splenectomy to be avoided or postponed in three patients. These very young children seem to have tolerated splenectomy with no OPSI when they were subjected to the same as older children. Five patients are considered lost to follow-up as their return visit to our clinic has been more than 5 years ago.

### Rapamycin (sirolimus)

Rapamycin, an mTOR inhibitor, has been used successfully in ALPS patients as first reported by Teachey et al. (23). ALPS-FAS patients with enlarged lymph nodes and spleen may require and benefit from rapamycin. Their cytopenias are usually refractory. Most ALPS patients on rapamycin show a good response in terms of reduction of lymphoproliferation as lymph nodes and spleen often shrink significantly. Many of the biomarkers including the signature DNT cells, serum IL10, serum vitamin B12 as well as HDL values normalize (20). Patients should be started with the initial loading dose of sirolimus (3 mg/m2), they can then be maintained with a dose of 2.5 mg/m2 given once a day (up to a maximum daily dose of 4 or 5 mg). Twenty-four-hour trough drug level of 5–15 ng/mL should be the target. Younger children may need a more frequent Q12 hourly dosing as they metabolize sirolimus more briskly than adults. It is critical to monitor for toxic side effects of sirolimus on kidney and liver function, and be wary of T-cell immunosuppression, hypercholesterolemia, and stomatitis. So far, we have used sirolimus in 14 individuals in our cohort, including mostly ALPS-FAS (*n* = 12) patients as a long-term steroid sparing measure. Their median age at treatment initiation has been 2 years (range 1–37 years) with a treatment duration spanning 6 months to 6 years. Four patients have discontinued therapy due to ineffectiveness of sirolimus and two adult patients have been weaned off of all the medications including rapamycin successfully due to resolution of their cytopenias.

### Rituximab

Rituximab (375 mg/m2 per week × 4) has also been used in 26 ALPS patients in our cohort as a treatment for their cytopenias. Most of them have received these treatments at outside hospitals, many before even ALPS could be suspected as a possible diagnosis. Sixteen patients responded with stable counts for at least 6 months and seven of them with ITP had responses lasting from 1 to 4 years. Children treated with rituximab for AIHA (*n* = 3) did not respond. Noted toxicities included persistent neutropenia, long lasting severe hypogammaglobulinemia. Many of these side effects have lasted for more than 5 years after the rituximab infusions in some of our ALPS-U patients. In our experience, rituximab-induced prolonged hypogammaglobulinemia compounds the risk of OPSI in asplenic individuals. We do not use rituximab in ALPS patients until all other options are exhausted (12).

### Hydroxychloroquine

There is always a role in select patients for use of old fashioned yet familiar medications like hydroxychloroquine, Dapsone, Azathioprine, or 6mercaptopurine for steroid sparing purposes. We have successfully managed two young female patients with hydroxychloroquine alone at doses 200–400 mg once daily as a single agent for refractory cytopenias and avoided long-term use of corticosteroids and other immunosuppressive agents for up to 7 years.

### TPO mimetic agents

Both eltrombopag (Promacta) and Nplate (Romiplostim) have been used in four of our patients. As highlighted in Table 1, two of them with thrombocytopenia responded with long-term remission for up to 4 years while on Promacta or Romiplostim and the other two did not respond. One of them continues to require both Promacta and MMF to maintain his platelet counts in a safe range of 50–100,000.

**Figure 1:**
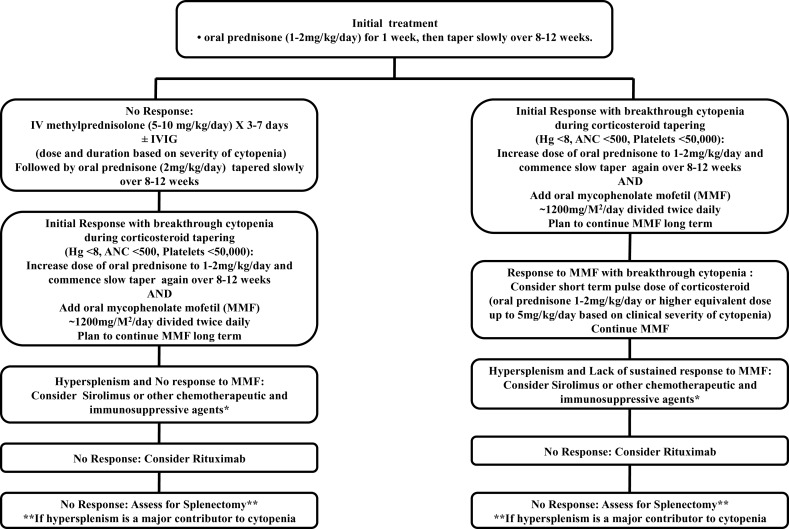
**This schematic diagram is only a suggested guideline for managing children with ALPS-associated autoimmune multilineage cytopenias and is derived from our published work (10)**. Use of G-CSF may be warranted for isolated neutropenia associated with systemic infections. Similarly use of other chemotherapeutic and immunosuppressive agents (e.g., vincristine, methotrexate, mercaptopurine, azathioprine, cyclosporine, hydroxychloroquine) besides mycophenolate mofetil (MMF) and sirolimus (rapamycin) can also be considered as a steroid sparing measure. This approach can be used for avoiding or at least postponing surgical splenectomy at the discretion of the treating clinicians based on the circumstances of a specific patient.

Use of short bridging courses of corticosteroids in ALPS patients, stabilizes their blood counts before commencing long-term single agent therapy with MMF, sirolimus, or hydroxychloroquine. One should not rely on them as a first-line upfront single agent treatment for significant cytopenias. MMF or sirolimus should be added to the therapy while they are being slowly tapered off their corticosteroids. Treatment with these agents should be commenced concomitantly with steroids within a 2–4 weeks period and dose adjusted over an 8- to 12-week period while the corticosteroids are being tapered with periodic monitoring of CBC. Management algorithm for approaching treatment decisions in ALPS patients is suggested in Figure 1 based on our experience highlighted above and is modified from our previous publication (10).

## Discussion

Many individual ALPS patients have to be exposed to long-term immunosuppression to remain free from their refractory cytopenias. Risk–benefit ratio should be assessed before exposing any patient to medications that might have long-term comorbidities. No significant iatrogenic end organ toxicities have been noted in any of our patients who have followed with us while receiving chronic therapy with MMF or sirolimus over the last 15 years. We have not used prophylaxis for fungal infections or pneumocystis jiroveci with fluconazole, septran (Bactrim), or pentamidine in those patients that have been maintained successfully on long-term therapy with MMF or sirolimus. There may be a role for long-term use of thrombopoietin mimetic agents and histone deacetylase inhibitors for managing recalcitrant cytopenias and hypersplenism. Novel and non-toxic targeted lympholytic therapies may become available in future to control the lymphoproliferative processes resulting in cytopenias in children with inherited disorders of immune function.

Continued search for new genetic mutations in the subgroup of ALPS patients with undetermined genetic defects (ALPS-U) are underway at our institution using emerging genomic and cell biology tools, including whole exome and genome sequencing and analysis. Some novel immune dysregulatory syndromes have been identified recently, leading to validation of candidate genes (e.g., RAS, CTLA4, LRBA, PI3Kinase, MagT1, STAT3-activating mutations, etc.) in their pathogenesis (25–31). Each of these syndromes is unique in some of their clinical presentations, while there is considerable overlap in their presentation with ALPS and common variable immune deficiency (CVID) (30, 32). The inherited genetic defect in them is not going to dissipate any time soon and hence many of them shall require similar long-term approaches for managing their clinical complications including autoimmune cytopenias as well as nodal and extranodal inflammatory and infiltrative lymphoid lesions leading to other end organ damage.

## Conclusion

Pediatric patients with refractory autoimmune cytopenias despite their normal bone marrows often challenge our skills in both diagnosis and management. Patients with ALPS and related disorders presenting with cytopenias often require long-term immunosuppressive therapies and consistent follow-up under care givers who are familiar with them. Vigilance toward the toxic side effects associated with each chosen therapeutic agent is imperative. These children present unique challenges and should be managed by harmonizing their diagnosis and care, including genetic counseling as needed for these emerging novel immunodysregulatory disorders. These approaches have implications in terms of resource allocation within the health care systems as the children of today transition into adolescents and young adults of tomorrow. All of them require monitoring and follow-up by multiple subspecialists familiar with their condition. It is desirable to not only utilize existing registries like ESID and USIDNET but also develop international registries beyond Europe and North America for rare and complex immune problems and chronic multilineage cytopenias in order to improve their management by elucidating and sharing their natural history while choosing rational therapies with local context.

## Conflict of Interest Statement

The author declares that the research was conducted in the absence of any commercial or financial relationships that could be construed as a potential conflict of interest.

## Footnotes

^1^http://www3.niaid.nih.gov/topics/ALPS/
